# Surprisingly Fast Interface and Elbow Angle Dynamics of Antigen-Binding Fragments

**DOI:** 10.3389/fmolb.2020.609088

**Published:** 2020-11-24

**Authors:** Monica L. Fernández-Quintero, Katharina B. Kroell, Martin C. Heiss, Johannes R. Loeffler, Patrick K. Quoika, Franz Waibl, Alexander Bujotzek, Ekkehard Moessner, Guy Georges, Klaus R. Liedl

**Affiliations:** ^1^Center for Molecular Biosciences Innsbruck (CMBI), Institute of General, Inorganic and Theoretical Chemistry, University of Innsbruck, Innsbruck, Austria; ^2^Roche Pharma Research and Early Development, Large Molecule Research, Roche Innovation Center Munich, Penzberg, Germany; ^3^Roche Pharma Research and Early Development, Large Molecular Research, Roche Innovation Center Zurich, Schlieren, Switzerland

**Keywords:** V_*H*_–V_*L*_ interface dynamics, C_*H*_1–C_*L*_ dynamics, elbow angle, antibody structure design, antibody structure prediction

## Abstract

Fab consist of a heavy and light chain and can be subdivided into a variable (V_*H*_ and V_*L*_) and a constant region (C_*H*_1 and C_*L*_). The variable region contains the complementarity-determining region (CDR), which is formed by six hypervariable loops, shaping the antigen binding site, the paratope. Apart from the CDR loops, both the elbow angle and the relative interdomain orientations of the V_*H*_–V_*L*_ and the C_*H*_1–C_*L*_ domains influence the shape of the paratope. Thus, characterization of the interface and elbow angle dynamics is essential to antigen specificity. We studied nine antigen-binding fragments (Fab) to investigate the influence of affinity maturation, antibody humanization, and different light-chain types on the interface and elbow angle dynamics. While the CDR loops reveal conformational transitions in the micro-to-millisecond timescale, both the interface and elbow angle dynamics occur on the low nanosecond timescale. Upon affinity maturation, we observe a substantial rigidification of the V_*H*_ and V_*L*_ interdomain and elbow-angle flexibility, reflected in a narrower and more distinct distribution. Antibody humanization describes the process of grafting non-human CDR loops onto a representative human framework. As the antibody framework changes upon humanization, we investigated if both the interface and the elbow angle distributions are changed or shifted. The results clearly showed a substantial shift in the relative V_*H*_–V_*L*_ distributions upon antibody humanization, indicating that different frameworks favor distinct interface orientations. Additionally, the interface and elbow angle dynamics of five antibody fragments with different light-chain types are included, because of their strong differences in elbow angles. For these five examples, we clearly see a high variability and flexibility in both interface and elbow angle dynamics, highlighting the fact that Fab interface orientations and elbow angles interconvert between each other in the low nanosecond timescale. Understanding how the relative interdomain orientations and the elbow angle influence antigen specificity, affinity, and stability has broad implications in the field of antibody modeling and engineering.

## Introduction

Antibodies are key players as therapeutic agents because of their ability to bind the majority of targets and their suitability for protein engineering (Chiu et al., 2019; Kaplon and Reichert, 2019; Kaplon et al., 2020). Description of the binding properties and characterization of the binding interface is essential for understanding the function of the antibody. The binding ability of antibodies is determined by the antigen-binding fragment (Fab), in particular the variable fragment region (Fv). The Fab consists of a heavy and a light chain and can be subdivided into two types of structurally distinct domains termed the variable (V_*H*_, V_*L*_) and constant domains (C_*H*_1, C_*L*_). The amino acid residues linking V_*L*_ to C_*L*_ and V_*H*_ to C_*H*_1 are called switch residues (Stanfield et al., 2006). In the antigen-binding process, the most important region is the complementarity-determining region (CDR), which consists of six hypervariable loops that shape the antigen-binding site, the paratope (Chothia et al., 1989; Martin and Thornton, 1996; Al-Lazikani et al., 2000; North et al., 2011). Apart from the diversity in length, sequence, and structure of the CDR loops, the relative V_*H*_–V_*L*_ interdomain orientation plays an important role in determining the shape of the antigen-binding site (Colman, 1988; Foote and Winter, 1992; Dunbar et al., 2013; Bujotzek et al., 2016). Various studies observed that mutations in the framework regions, in particular in the V_*H*_–V_*L*_ interface, can strongly influence the antigen-binding affinity. Thus, mutations in the V_*H*_–V_*L*_ interface result in structural changes of the binding site geometry, thereby modifying the relative V_*H*_–V_*L*_ orientation (Riechmann et al., 1988; Foote and Winter, 1992; Braden et al., 1994; Banfield et al., 1997; Cauerhff et al., 2004). Numerous studies in literature focused on defining this relative interdomain orientation (Narayanan et al., 2009; Abhinandan, 2010; Almagro et al., 2011; Chailyan et al., 2011). The most commonly used and robust approach to characterize the V_*H*_–V_*L*_ pose is ABangle (Dunbar et al., 2013; Teplyakov et al., 2014; Bujotzek et al., 2015, 2016). ABangle is a computational tool to characterize the relative orientations between the antibody variable domains (V_*H*_ and V_*L*_) by using five angles and a distance and by comparing it to other known structures (Dunbar et al., 2013; Bujotzek et al., 2015, 2016).

The high variability in the V_*H*_–V_*L*_ interdomain orientation is an additional feature of antibodies, which directly increases the size of the antibody repertoire (Chothia et al., 1985; Vargas-Madrazo and Paz-García, 2003; Bujotzek et al., 2016; Knapp et al., 2017; Fernández-Quintero et al., 2020c). This high variability in the V_*H*_–V_*L*_ interdomain distribution has been reported for different IL-1β antibody fragments in agreement with the respective NMR ensembles (Fernández-Quintero et al., 2020c). By applying fast Fourier transformation to the interface angles, timescales of 0.1–10 GHz could be assigned to the fastest collective interdomain movements (Fernández-Quintero et al., 2020c). With the increasing number of available Fab X-ray structures, it was noted that these fragments also display a high variability in the elbow angle, which is defined as the angle between the pseudo-two-fold axes relating V_*H*_ to V_*L*_ and C_*H*_1 to C_*L*_ (Sotriffer et al., 2000; Stanfield et al., 2006). The elbow angle has been shown to increase Fab flexibility and thereby to enhance the ability of the same antibody to recognize different antigens (Landolfi et al., 2001; Stanfield et al., 2006; Niederfellner et al., 2011). Additionally, it has been shown that mutations in the Fab elbow region can influence the interdomain conformational flexibility and paratope plasticity (Sotriffer et al., 2000; Henderson et al., 2019).

The C_*H*_1–C_*L*_ heterodimer was found to be significantly more stable than the V_*H*_–V_*L*_ heterodimer and has been shown to play an essential role for antibody assembly and secretion in the cell (Röthlisberger et al., 2005; Bönisch et al., 2017). Mutual stabilization occurred across both Fab interfaces, and a high degree of cooperation between V_*H*_–V_*L*_ and C_*H*_1–C_*L*_ could be observed. However, direct interactions among each domain (V_*L*_, C_*L*_/V_*H*_, and C_*H*_1) did not influence the stability of either domain (Röthlisberger et al., 2005).

In this study, we investigate the dynamics of both relative V_*H*_–V_*L*_, C_*H*_1–C_*L*_ interface angles and the elbow angle and their respective dependencies on different light-chain types and shifts upon antibody humanization and affinity maturation. The aim is to structurally and mechanistically characterize these interdomain movements and elbow angle flexibilities and assign and estimate timescales to these domain motions.

## Materials and Methods

### Investigated Antibody Fabs

The nine investigated publicly available Fab X-ray structures were chosen to have a representative set of antibodies covering various challenges in antibody engineering and design, as they differ in light-chain types (PDB accession codes: 1PLG, 1NL0, 1BBD, 7FAB, and 1DBA), upon humanization (PDB accession codes: 3L7E, 4PS4) and affinity maturation (1MLB, 2Q76).

### Structure Preparation

All Fab X-ray structures were prepared in MOE (Molecular Operating Environment, Montreal, QC, Canada: 2019) (Molecular Operating Environment [MOE], 2020) using the Protonate 3D (Labute, 2009) tool. With the tleap tool of the Amber Tools20 package, the Fab structures were placed into cubic water boxes of TIP3P (Jorgensen et al., 1983) water molecules with a minimum wall distance to the protein of 10 Å (El Hage et al., 2018; Gapsys and de Groot, 2019). Parameters for all antibody simulations were derived from the AMBER force field 14SB (Maier et al., 2015). To neutralize the charges, we used uniform background charges (Darden et al., 1993; Salomon-Ferrer et al., 2013; Hub et al., 2014). Each system was carefully equilibrated using a multistep equilibration protocol (Wallnoefer et al., 2011).

All Fabs were simulated twice for 1 μs with different initial velocities, using molecular dynamics as implemented in the AMBER 20 (Case et al., 2020) simulation package. The results for the second 1 μs simulations are summarized in Supplementary Table 1, as the conclusions are the same as for the simulations presented in the manuscript. We removed the equilibration and relaxation phase in the respective simulations. Molecular dynamics simulations were performed using pmemd.cuda (Salomon-Ferrer et al., 2013) in an NpT ensemble to be as close to the experimental conditions as possible and to obtain the correct density distributions of both protein and water. Bonds involving hydrogen atoms were restrained by applying the SHAKE algorithm (Miyamoto and Kollman, 1992), allowing a time step of 2.0 fs. Atmospheric pressure of the system was preserved by weak coupling to an external bath using the Berendsen algorithm (Berendsen et al., 1984). The Langevin thermostat was used to maintain the temperature at 300 K during simulations (Adelman and Doll, 1976).

### Interface Angle Calculations

ABangle is a computational tool (Dunbar et al., 2013; Bujotzek et al., 2015, 2016; Fernández-Quintero et al., 2020c) used to characterize the relative orientations between the antibody variable domains (V_*H*_ and V_*L*_) using six measurements (five angles and a distance). A plane is projected on each of the two variable domains. To define these planes, the first two components of a principal component analysis of 240 reference coordinates were used for V_*H*_ and V_*L*_ each. The reference coordinate set consists of Cα coordinates of eight conserved residues for 30 cluster representatives from a sequence clustering of the non-redundant ABangle antibody data set. The planes were then fit through those 240 coordinates, and consensus structures consisting of 35 structurally conserved Cα positions were created for the V_*H*_ and V_*L*_ domain. Between these two planes, a distance vector C is defined. The six measures are then two tilt angles between each plane (HC1, HC2, LC1, and LC2) and a torsion angle (HL) between the two planes along the distance vector C (dc). The ABangle script can calculate these measures for an arbitrary Fv region by aligning the consensus structures to the found core set positions and fitting the planes and distance vector from this alignment. This online available tool was combined with an in-house python script to reduce computational effort and to visualize our simulation data over time. The in-house script makes use of ANARCI (Dunbar and Deane, 2016) for fast local annotation of the Fv region and pytraj for rapid trajectory processing. The resulting fluctuations in the HL angle (Supplementary Figure S3) were further analyzed with a fast Fourier transformation (FFT) (Bergland, 1969) in python to characterize the frequency and timescale of these movements. We applied a frequency filter to assign timescales to movements.

To characterize the relative interdomain C_*H*_1 and C_*L*_ orientations (Supplementary Figure S3), we defined a torsion angle between the center of mass (COM) of the loops of the C-terminal C_*H*_1 domain, the COM of the C_*H*_1, the COM of the C_*L*_ domain, and the COM of the loops of the C-terminal C_*L*_ domain.

**FIGURE 1:**
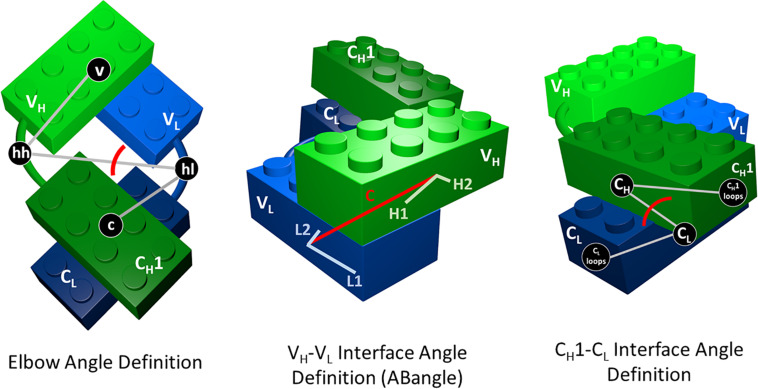
Elbow angle, ABangle (HL angle), and C_*H*_1–C_*L*_ interface angle definitions depicted as Lego models. The constant domains are illustrated in dark green (C_*H*_1) and dark blue (C_*L*_), while the variable domains are shown in light green (V_*H*_) and light blue (V_*L*_). To calculate the C_*H*_1–C_*L*_ interface angle, we defined a torsion angle between the center of mass of the C-terminal loops of the C_*H*_1 domain, the center of mass of C_*H*_1, the center of mass of the C_*L*_ domain, and the center of mass of the C-terminal loops of the C_*L*_ domains. The center of masses of the C_*H*_1 and C_*L*_ C-terminal loops are depicted in this figure, as C_*L*_ and C_*H*_1 loops. For the elbow angle definition, all used centers of mass to define the torsion angle are illustrated. The centers of mass of the switch or hinge regions are abbreviated with hh (hinge heavy) and hl (hinge light).

As measure for the elbow angle (Supplementary Figure S3), we calculated a torsion angle between the COM of the variable domain, a defined vector between the COMs of the switch regions (hinge heavy and hinge light) and the COM of the constant region. Figure 1 depicts all used interface and elbow angle definitions, showing the Fab domains as Lego model.

## Results

The first five introduced antigen-binding fragments are part of a study, discussing the influence of different light-chain types (κ and λ light chains) on the resulting elbow angle distributions observed in X-ray structures (Stanfield et al., 2006). While the other six discussed Fabs contribute to a better understanding of the interface and elbow angle flexibilities upon antibody humanization and affinity maturation (Cauerhff et al., 2004; Fransson et al., 2010). By using MD simulations, we investigate the conformational variability of these interface and elbow angle distributions in solution and assign timescales to the dynamics of these movements, which have direct implications in the design of antibody paratopes and molecular recognition. The first investigated antibody is the 10C12 antibody (PDB accession code: 1NL0), inhibiting the human Factor IX calcium-stabilized N-terminal gamma-carboxyglutamic acid-rich (Gla) domain, which is a membrane-anchoring domain found on vitamin K-dependent blood coagulation and regulatory proteins. The 10C12 antibody is a conformation-specific anti-Factor IX antibody to interfere with the Factor IX-membrane interaction (Huang et al., 2004). Same as the 10C12 antibody, the highly resolved IgG1 Fab structure with the PDB accession code 7FAB also contains a λ light chain. The biggest difference between the two Fab structures is the elbow angle orientation.

**FIGURE 2:**
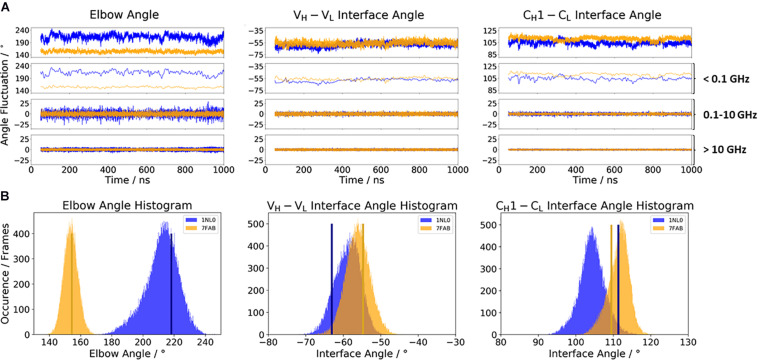
**(A)** Comparison of the two considered λ light-chain antibodies 10C12 (PDB: 1NL0) and IgG1 Fab (PDB: 7FAB). The biggest difference between these two antibodies is the elbow angle. The distributions of the 10C12 antibody are depicted in blue, while the IgG1 antibody angle distributions are colored yellow. Additionally, the FFT of the respective distributions are displayed, showing the angle variations occurring faster than 1 ns, between 0.1 and 10 ns, and slower than 10 ns. **(B)** Histograms of the respective interface and elbow angle distributions, including the respective X-ray structures of both λ light chain antibodies, which were used as starting structures for molecular dynamics simulations.

**FIGURE 3:**
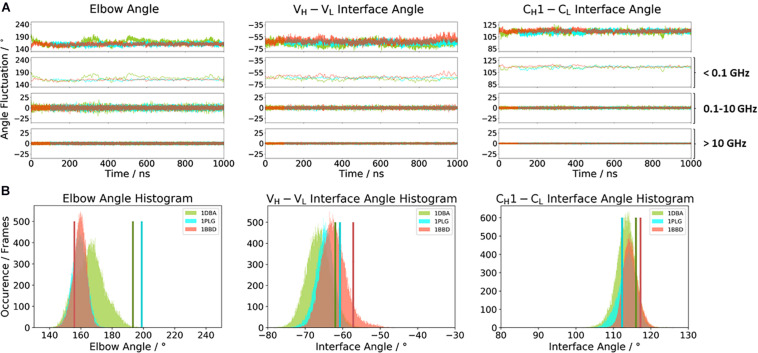
**(A)** Comparison of the three κ light chain antibodies, which also show a big spread in the elbow angle in the crystal structures. The specific anti-progesterone antibody DB3 (PDB: 1DBA) is colored in green. The 8F5 antibody (PDB: 1BBD) is colored in red, and the X-ray structure of the murine antibody which has a high specificity toward α-(2→8)-linked sialic acid polymers (PDB: 1PLG) is colored light blue. Additionally, the FFT of the respective distributions are displayed, showing the angle variations occurring faster than 1 ns, between 0.1 and 10 ns, and slower than 10 ns. **(B)** Histograms of the respective interface and elbow angle distributions, including the respective X-ray structures of all three κ light chain antibodies, which were used as starting structures for molecular dynamics simulations.

Figure 2A illustrates the respective distributions and the results of the fast Fourier transformation (FFT) of the two λ light-chain antibodies for both interface angles (V_*H*_–V_*L*_ and C_*H*_1–C_*L*_) and the elbow angle. The 10C12 antibody is colored in blue, while the IgG1 7FAB antibody is colored yellow. The fast Fourier transformation shows that all angles of both the 10C12 and IgG1 7FAB antibodies have high variations and allows to assign timescales of 0.1–10 GHz to the fastest collective angle movements. The highest flexibility and variability can be observed for the elbow angle, which fluctuates about ±15° in less than 10 ns, while both interface angles fluctuate around ±5° in less than 1 ns. Especially interesting is that these fast fluctuations in the low nanosecond timescale are substantially faster compared to conformational rearrangements in the antibody paratope, which is in line with previous studies (Fernández-Quintero et al., 2019a, c, 2020a,b). Additionally, also from the histograms (Figure 2B) it can be seen that the elbow angle has the highest variability, compared to the interface angle distributions. The starting X-ray structures of the respective antibodies are plotted into the histograms and color-coded, respectively. The third antibody investigated is the highly specific anti-progesterone antibody DB3 (PDB accession code: 1DBA) which can bind progesterone with nanomolar affinity. The DB3 antibody (containing a κ light chain) binds progesterone by forming a hydrophobic pocket by interactions between the three complementarity determining regions L1, H2, and H3 (Arevalo et al., 1993). Another example for a κ light-chain antibody is the IgG2 κ murine monoclonal antibody with high specificity for α-(2→8)-linked sialic acid polymers (PDB accession code 1PLG) (Evans et al., 1995). The fifth studied antigen-binding fragment (IgG2, κ light chain) 8F5, which is obtained by immunization with the native HRV2, neutralizes human rhinovirus serotype 2 and cross-reacts with peptides of the viral capsid protein VP2 (PDB accession code 1BBD) (Tormo et al., 1992). All three κ light-chain antibodies were simulated for two times 1 μs, and the results are depicted in Figure 3. In line with the results of the λ light-chain Fabs, the FFT in Figure 3A shows that the variability of the interface and elbow angles can be captured in the low nanosecond timescale. The histograms in Figure 3B clearly show that compared to Figure 2, especially in the elbow angle and the C_*H*_1–C_*L*_ interface histograms, the distributions have much more overlay and are also narrower, indicating less variability and diversity in these angles when considering κ light-chain antibodies.

**FIGURE 4:**
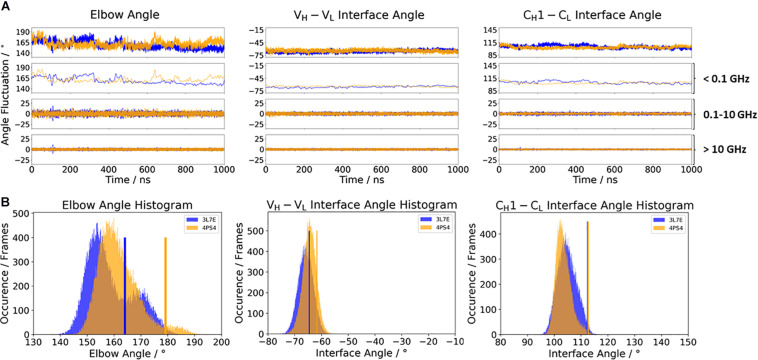
**(A)** Influence of different framework origins on the relative interface and elbow angle distributions. The chimeric c836 antibody (PDB: 3L7E) is illustrated in blue, while the optimized humanization variant m1295 (PDB: 4PS4) is depicted in orange. The calculations of the FFT of the respective distributions are displayed, showing the angle variations occurring faster than 1 ns, between 0.1 and 10 ns, and slower than 10 ns. **(B)** Histograms of the respective interface and elbow angle distributions, including the respective X-ray structures of the chimeric and the humanized Fabs, which were used as starting structures for molecular dynamics simulations.

To investigate the effect of antibody humanization, we chose the humanization of a mouse anti-human IL-13 antibody (PDB accession codes: 3L7E and 4PS4) (Fransson et al., 2010; Teplyakov et al., 2011). The antibodies are humanized by the human-framework adaptation method (HFA), which comprises a selection (human framework selection), and an optimization (specificity-determining residue optimization) step. IL-13 is an important member of the growth-hormone-like cytokine family and is involved in the development of asthma (Grünig et al., 2012). Figure 4 shows the comparison of the c836 antibody with the humanized Specificity Determining Residue Optimization (SDRO) optimized m1295 Fab to investigate if the relative interdomain orientations and the elbow angle distributions are shifted upon antibody humanization. While the relative interdomain V_*H*_–V_*L*_ angle distributions are slightly shifted, the C_*H*_1–C_*L*_ interface angle distribution for the m1295 variant completely overlaps with the c836 Fab and is much narrower, as a result of the specificity optimization process (Figure 4A). The elbow angle distribution for the chimeric c836 Fab is shaped bimodally, while m1295 has only one dominant elbow angle minimum in solution (Figure 4B). Again, the variability of the interface and elbow angle movements can be captured, as their fluctuations occur in the 0.1–10 GHz timescale.

**FIGURE 5:**
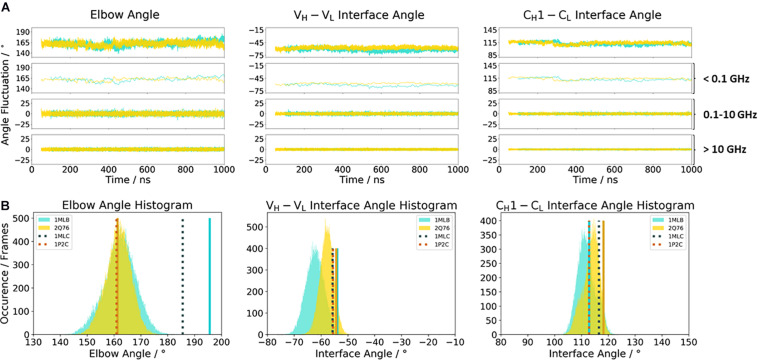
**(A)** Influence of affinity maturation on the relative interface and elbow angle distributions. The D44.1 Fab (colored in cyan) reveals a broader interface and elbow angle distribution compared to the matured F10.6.6 antibody. The calculations of the FFT of the respective distributions are displayed, showing the angle variations occurring faster than 1 ns, between 0.1 and 10 ns, and slower than 10 ns. **(B)** Histograms of the respective interface and elbow angle distributions, including the respective X-ray structures of the affinity maturation pair D44.1 and F10.6.6, which were used as starting structures for molecular dynamics simulations.

Another unique ability of antibodies is to evolve in response to antigens and undergo cycles of mutation and selection leading to an enhanced affinity and specificity (Wabl and Steinberg, 1996; Acierno et al., 2007; Mishra and Mariuzza, 2018). To understand and characterize the underlying biophysical mechanism of affinity maturation, we investigated the maturation of an anti-chicken egg-white lysozyme antibody D44.1 (PDB accession codes 1MLB and 2Q76) (Braden et al., 1994; Cauerhff et al., 2004). Both D44.1 and the matured F10.6.6 Fab are murine monoclonal antibodies, which are related in sequence and structure as they origin from the same gene rearrangement. The affinity matured F10.6.6 antibody (K_*A*_ = 1.02^∗^10^10^ M^–1^) was reported to have a 700-times higher-affinity constant compared to D44.1 (K_*A*_ = 1.44^∗^10^7^ M^–1^), due to a higher surface complementarity to the antigen (Acierno et al., 2007). The D44.1 Fab differs from the affinity matured variant F10.6.6 in twenty mutations, seven of them located in the CDR loops, while the other mutations can be found in both the V_*H*_–V_*L*_ and C_*H*_1–C_*L*_ interface. As the majority of mutations occur in the framework, already on the structural level a stabilization of the V_*H*_–V_*L*_ interface has been reported (Braden et al., 1994; Cauerhff et al., 2004). Figure 5 shows the angle distributions of the D44.1 antibody compared to the further matured F10.6.6 antibody. Upon affinity maturation, we observe a rigidification in the V_*H*_–V_*L*_ angle and elbow angle distributions (Figure 5A). This rigidification can also be confirmed by the narrower histograms of the matured F10.6.6 Fab illustrated in Figure 5B. In agreement with previous results, the FFT of both the D44.1 and F10.6.6 antibodies shows that also in this example the dynamics and flexibility of the interface and elbow angle distributions occur in the low nanosecond timescale. We used the X-ray structures crystallized without antigen as starting structure for the simulations to identify whether the binding competent relative interdomain and elbow angle orientations are preexisting without the presence of the antigen. We clearly see that for the D44.1 antibody the relative interdomain orientation of the crystal structure binding to the antigen is present and more favorable in solution compared to the X-ray structure without the antigen. The resulting elbow angle distribution (Figure 5B) in solution shows that none of the two available crystal structures of the D44.1 antibody is actually favored in solution. Upon maturation, the relative interdomain orientations, especially the V_*H*_–V_*L*_ orientation, in the X-ray structures do not change anymore upon binding, which is in line with the observed rigidification already on the X-ray structural level. The fact that we sample all binding competent V_*H*_–V_*L*_ interface orientations supports the idea of a preexisting conformational ensemble out of which the binding competent state is selected and therefore follows the paradigm of conformational selection (Ma et al., 1999; Tsai et al., 1999; Fernández-Quintero et al., 2019a, b, 2020e).

## Discussion

In this present study, we characterize and quantify the relative interdomain and elbow angle orientations between antibodies bearing κ or λ light chains and between antibodies before and after humanization, upon affinity maturation. By using FFT, we were able to assign timescales to these fast interface and elbow angle movements in the low nanosecond timescale, which has direct implications in the field of antibody structure engineering and design.

Various studies already investigated the influence of different light chains (κ or λ light chains) on phenotypic differences, e.g., conformational flexibility, half-life, and propensity to alter antibody specificity (Montaño and Morrison, 2002; Wardemann et al., 2004; Stanfield et al., 2006; Townsend et al., 2016). Thus, the differences in κ and λ light chains result in distinct binding specificities. In line with previous observations, we observe that κ and λ light chains differ in their conformational flexibility. While the distributions in interface and elbow angles of the κ light-chain antibodies—independently of their starting geometries—overlap with each other and result in similar favorable orientations in solution, the Fabs consisting of a λ light chain reveal shifts and a higher diversity in possible elbow angles and interface orientations (Figures 2, 3). We can clearly see from the FFT that the fast interface and elbow angle movements take place in the low nanosecond timescale (0.1–10 GHz) independent of the light chain (Figures 2A, 3A). We particularly chose the antibodies to have the biggest spread in the elbow angle orientations, ranging from 127° to 220° (Supplementary Figure S1). The 10C12 antibody (Figure 2A—blue) shows overall much more variability in all interface and elbow angles in the 0.1–10 GHz timescale, compared to the IgG1 7FAB antibody.

**FIGURE 6:**
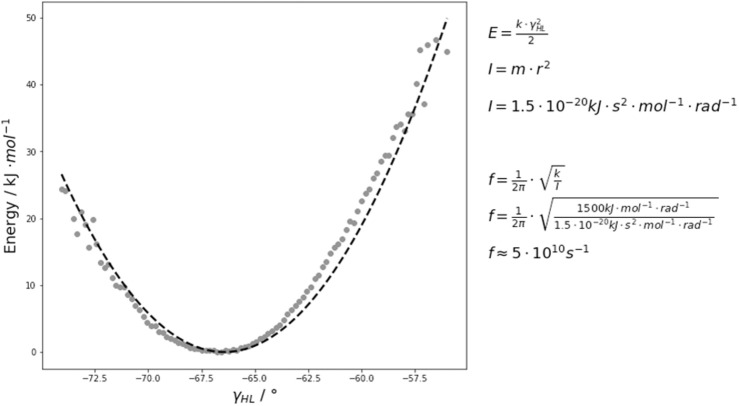
An exemplary free energy surface of the DB3 antibody with the fitted quadratic function is illustrated and shows that these interdomain and elbow angle fluctuations interconvert with each other in the 0.1–10 GHz timescale. We estimated the force constants k and included the respective equations used for the frequency f calculations. The variable I represents the moment of inertia which was used to calculate the frequencies.

The free energy surfaces of the interface and elbow angle movements are shaped parabolically. Thus, if the fast movements of the interface and elbow angle are approximated by a harmonic potential, the force constants by fitting the free energy curves to quadratic functions and calculated the characteristic frequencies of the domain movements by using classical mechanics. As observed by the FFT, the majority of the interdomain and elbow angle dynamics occurs in the low nanosecond timescale (Figure 6 and Supplementary Figure S4). Figure 6 illustrates the respective free energy surface with the fitted quadratic functions. The fluctuations of these interdomain and elbow angles occur in the 0.1–10 GHz timescale and interconvert between each other in the 0.1–10 GHz timescale. The fact that these interface and elbow angles fluctuate ±5°/±10° within this single minimum in solution introduces a new view on these interfaces which directly influences the design and structure prediction of antibodies. Compared to the fast interdomain and elbow angle dynamics, the loop rearrangements occur in the high micro-to-millisecond timescale. Therefore, changes in the CDR loop conformations might be responsible for the dynamics slower than 10 ns. Thus, also conformational changes of the paratope directly influence the relative interdomain orientations and the elbow angle (Sotriffer et al., 1998; Sotriffer et al., 2000; Fernández-Quintero et al., 2020c, f).

In the context of antibody humanization (Zhang et al., 2013; Margreitter et al., 2016), apart from the CDR loop length and sequence, the relative V_*H*_–V_*L*_ interdomain orientation has already been discussed to directly influence antigen binding (Bujotzek et al., 2016). Modulation of the V_*H*_–V_*L*_ orientation diversifies antibody paratopes and thereby allows to accommodate diverse antigenic shapes that antibodies are confronted with (Teplyakov et al., 2011; Bujotzek et al., 2016). Figure 4 shows the humanization of a mouse anti-human IL-13 antibody, which after the humanization and SDRO process showed a higher specificity compared to the murine (Fransson et al., 2010). This step-by-step antibody humanization has already been shown to result in a reduced conformational diversity, reflected by a substantial decrease in conformational space (Fernández-Quintero et al., 2020a). Our results are perfectly in line with these observations, as the C_*H*_1–C_*L*_ interface angle and the elbow angle rigidify upon humanization. Additionally, we were able to identify a small shift in the V_*H*_–V_*L*_ interface distribution in solution for the m1295, which might be more favorable and contribute to better recognition and binding of the antigen.

Elucidating the affinity maturation process has been the focus of numerous studies (Cauerhff et al., 2004; Cho et al., 2005; Acierno et al., 2007; Li et al., 2010; Wong et al., 2011; Adhikary et al., 2015; Jeliazkov et al., 2018; Mishra and Mariuzza, 2018; Fernández-Quintero et al., 2019b; Shehata et al., 2019; Chan et al., 2020). Upon affinity maturation (Figure 5), we observe for the matured F10.6.6 antibody in both V_*H*_–V_*L*_ interface and elbow angle histograms (Figure 5B) a narrower distribution, compared to the broader surface of the D44.1 Fab (Supplementary Table S1). A structural ensemble for both antibodies before and after affinity maturation is illustrated in Supplementary Figure S2, and also the rigidification upon affinity maturation is reflected in a lower number of clusters. Even though rigidification might only be one of the various consequences of affinity maturation, it still represents a fundamental mechanism resulting in an increase in specificity (Thorpe and Brooks, 2007; Li et al., 2015; Di Palma and Tramontano, 2017). Therefore, understanding the interface and elbow angle flexibility and dynamics upon affinity maturation is a prerequisite for all other affinity increasing changes, e.g., improved interfacial interactions, increased buried surface area, and improved shaped complementarity (Fernández-Quintero et al., 2020d, e). This observed rigidification, not only in the CDR loops but also in the V_*H*_–V_*L*_ and elbow angle dynamics, clearly confirms the role of the interdomain dynamics in tailoring antibody specificity. All binding competent interface and elbow angle orientations preexist in solution, without the presence of the antigen. Thus, the relative interdomain and elbow angles clearly follow the concept of conformational selection (Ma et al., 1999).

## Conclusion

For all investigated antibodies, we observe that changes in the sequences (e.g., different light-chain types, humanization, and affinity maturation) can influence and shift the interface and elbow angle distributions. Our results show that antibodies with a λ light chain do not only have broader X-ray angle distributions but also have higher variations in their relative interface angle distributions, especially in the C_*H*_1 and C_*L*_ distributions. Upon humanization of a mouse anti-human IL-13 antibody, we observe small shifts in the V_*H*_–V_*L*_ distributions and a rigidification in C_*H*_1 and C_*L*_ and elbow angle distributions. In line with the rigidification as a consequence of the specificity optimization process, we also observe a rigidification in the V_*H*_–V_*L*_ and elbow angle distributions upon affinity maturation. The rigidification upon affinity maturation might only be one of various consequences; however, understanding the flexibilities of the antibody interfaces is prerequisite for all other specificity-increasing changes. Both Fab interfaces and the elbow angle show movements occurring in the 0.1–10 GHz timescale (fluctuations around ±5°/±10°, respectively), which directly influence the binding site geometry. Thus, the understanding of these fast dynamics has broad implications in the field of antibody structure prediction and design.

## Data Availability Statement

The original contributions presented in the study are included in the article/Supplementary Material, further inquiries can be directed to the corresponding author.

## Author Contributions

The manuscript was discussed and written through contributions of all authors. All authors have given approval to the final version of the manuscript.

## Conflict of Interest

AB, EM, and GG were Roche employees: Roche has an interest in developing antibody-based therapeutics. The remaining authors declare that the research was conducted in the absence of any commercial or financial relationships that could be construed as a potential conflict of interest.
