# Supplementary material for: Surprisingly Fast Interface and Elbow Angle Dynamics of Antigen-Binding Fragments
Source: Front Mol Biosci. 2020 Nov 24;7:609088. doi: 10.3389/fmolb.2020.609088 (PMC7732698; doi:10.3389/fmolb.2020.609088)
Supplement: Supplementary file 1 [file Table_1.DOCX]

Supporting Information

SI Table S1: The Table shows the relative V_H_-V_L_ and C_H_1-C_L_ interface angle averages and standard deviations for all investigated systems.

V_H_-V_L_ Interface Angle

| PDB-Code | Simulation | µ / ° | σ / ° | min / ° | max / ° | Δ / ° |
| --- | --- | --- | --- | --- | --- | --- |
| 3L7E | 1 | -65.8 | 2.4 | -75.5 | -55.3 | 20.2 |
|  | 2 | -65.9 | 2.7 | -75.7 | -55.0 | 20.7 |
| 4PS4 | 1 | -64.3 | 2.7 | -75.0 | -52.7 | 22.3 |
|  | 2 | -64.5 | 2.5 | -76.7 | -53.4 | 23.3 |
| 1NL0 | 1 | -57.3 | 2.1 | -73.6 | -46.8 | 26.8 |
|  | 2 | -58.4 | 3.3 | -74.5 | -46.6 | 27.9 |
| 1PLG | 1 | -64.7 | 2.4 | -75.7 | -53.8 | 21.9 |
|  | 2 | -65.1 | 2.7 | -76.3 | -54.1 | 22.2 |
| 1DBA | 1 | -67.6 | 2.9 | -78.4 | -54.4 | 24.0 |
|  | 2 | -66.4 | 3.3 | -79.2 | -52.6 | 26.6 |
| 7FAB | 1 | -56.7 | 2.6 | -68.7 | -43.5 | 25.2 |
|  | 2 | -56.1 | 3.2 | -70.1 | -41.1 | 29.0 |
| 1BBD | 1 | -62.3 | 3.3 | -74.3 | -46.4 | 27.9 |
|  | 2 | -60.1 | 3.0 | -71.9 | -44.5 | 27.4 |
| 1MLB | 1 | -64.8 | 3.4 | -72.4 | -50.3 | 22.1 |
|  | 2 | -62.9 | 3.5 | -73.7 | -49.5 | 24.2 |
| 2Q76 | 1 | -58.2 | 2.4 | -51.7 | -67.4 | 15.7 |
|  | 2 | -59.0 | 2.4 | -49.3 | -65.8 | 16.5 |

C_H_1-C_L_ Interface Angle

| PDB-Code | Simulation | µ / ° | σ / ° | min / ° | max / ° | Δ / ° |
| --- | --- | --- | --- | --- | --- | --- |
| 3L7E | 1 | 104.7 | 3.3 | 92.7 | 120.7 | 28.0 |
|  | 2 | 104.8 | 3.5 | 93.4 | 117.1 | 23.7 |
| 4PS4 | 1 | 104.9 | 2.7 | 95.7 | 116.7 | 21.0 |
|  | 2 | 103.5 | 2.8 | 95.7 | 115.7 | 20.0 |
| 1NL0 | 1 | 91.4 | 3.6 | 79.8 | 104.6 | 24.8 |
|  | 2 | 104.2 | 3.2 | 91.7 | 117.7 | 26.0 |
| 1PLG | 1 | 113.6 | 2.4 | 103.6 | 121.7 | 18.1 |
|  | 2 | 99.7 | 4.7 | 83.9 | 115.9 | 32.0 |
| 1DBA | 1 | 109.7 | 3.2 | 98.5 | 124.0 | 25.5 |
|  | 2 | 113.3 | 2.6 | 99.8 | 126.2 | 26.4 |
| 7FAB | 1 | 110.9 | 3.3 | 95.4 | 123.6 | 28.2 |
|  | 2 | 111.4 | 2.8 | 99.0 | 122.2 | 23.2 |
| 1BBD | 1 | 114.5 | 1.9 | 106.3 | 122.6 | 16.3 |
|  | 2 | 114.2 | 2.1 | 104.8 | 123.1 | 18.3 |
| 1MLB | 1 | 111.3 | 3.0 | 101.7 | 125.4 | 23.7 |
|  | 2 | 112.7 | 3.3 | 100.9 | 125.8 | 24.9 |
| 2Q76 | 1 | 115.4 | 2.8 | 105.6 | 128.9 | 23.3 |
|  | 2 | 116.2 | 2.6 | 104.3 | 127.2 | 22.9 |

Elbow Angle

| PDB-Code | Simulation | µ / ° | σ / ° | min / ° | max / ° | Δ / ° |
| --- | --- | --- | --- | --- | --- | --- |
| 3L7E | 1 | 159.4 | 6.2 | 140.0 | 188.6 | 48.6 |
|  | 2 | 158.8 | 8.6 | 137.4 | 188.0 | 50.6 |
| 4PS4 | 1 | 162.8 | 10.3 | 138.8 | 203.0 | 64.2 |
|  | 2 | 161.7 | 7.5 | 143.7 | 194.8 | 51.1 |
| 1NL0 | 1 | 204.7 | 10.2 | 166.7 | 238.6 | 71.9 |
|  | 2 | 212.0 | 10.3 | 169.1 | 240.6 | 71.5 |
| 1PLG | 1 | 159.1 | 4.7 | 139.8 | 181.0 | 41.2 |
|  | 2 | 155.8 | 6.9 | 130.7 | 189.0 | 58.3 |
| 1DBA | 1 | 167.4 | 10.0 | 136.9 | 201.8 | 64.9 |
|  | 2 | 166.0 | 9.1 | 139.2 | 200.0 | 60.8 |
| 7FAB | 1 | 152.9 | 4.7 | 133.5 | 173.0 | 39.5 |
|  | 2 | 153.7 | 4.5 | 136.4 | 173.4 | 37.0 |
| 1BBD | 1 | 159.3 | 4.3 | 140.3 | 178.8 | 38.5 |
|  | 2 | 161.5 | 5.9 | 142.3 | 199.7 | 57.4 |
| 3PP3 – WT | 1 | 154.7 | 5.4 | 135.6 | 175.2 | 39.6 |
|  | 2 | 154.7 | 5.6 | 134.3 | 181.3 | 47.0 |
| 3PP3 – MUT | 1 | 156.3 | 6.1 | 137.1 | 182.1 | 45.0 |
|  | 2 | 158.8 | 9.1 | 137.2 | 192.8 | 55.6 |
| 1MLB | 1 | 157.9 | 4.8 | 138.4 | 179.7 | 41.3 |
|  | 2 | 158.2 | 4.7 | 139.8 | 182.5 | 42.7 |
| 2Q76 | 1 | 162.4 | 4.1 | 146.6 | 181.6 | 35.0 |
|  | 2 | 161.6 | 3.9 | 144.2 | 178.8 | 34.6 |


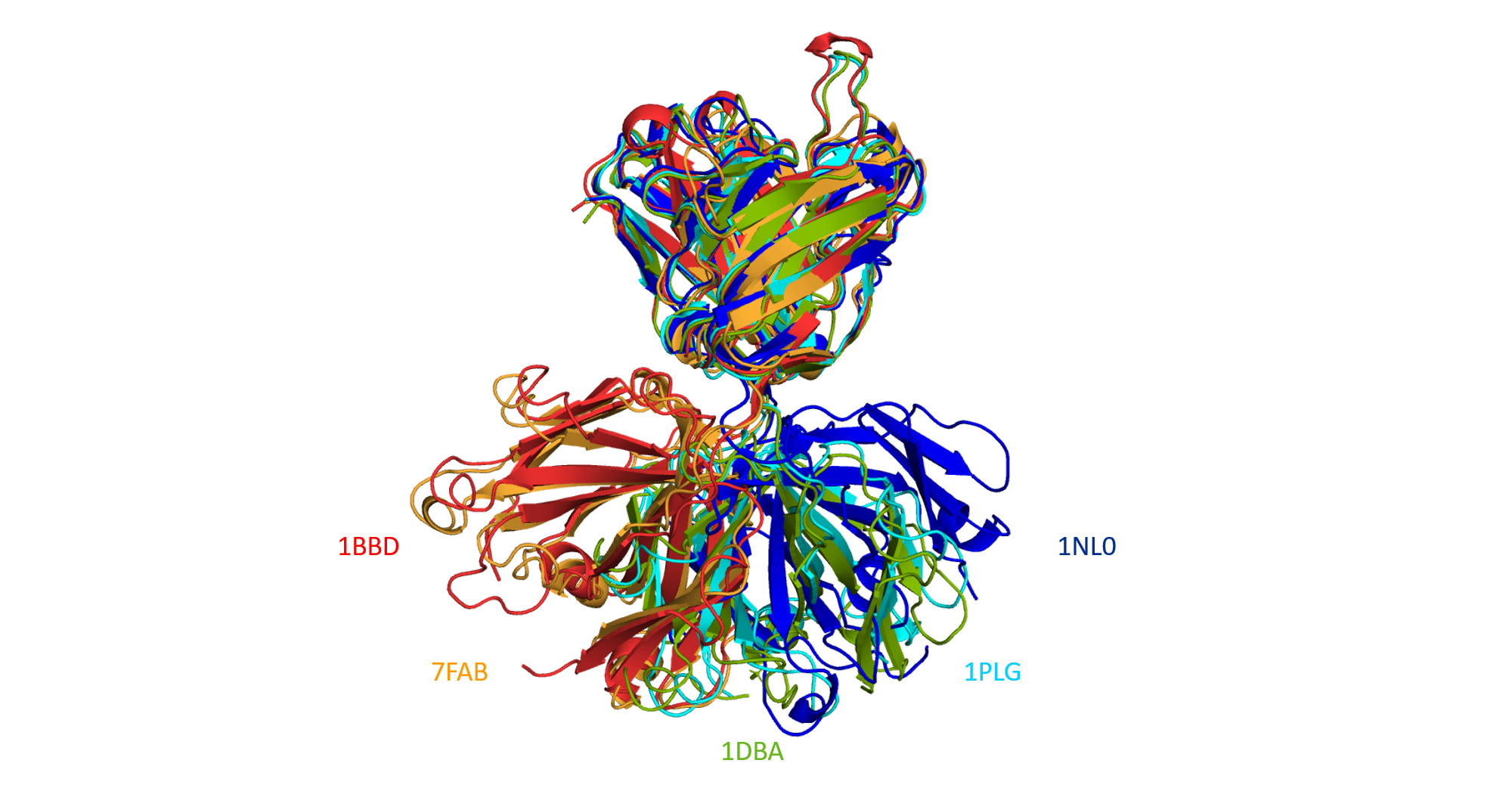


SI Figure S1: Overview of all investigated systems which differ substantially in their elbow angle orientations with the respective PDB accession codes. The color-coding also corresponds to the angle distributions presented in the manuscript.


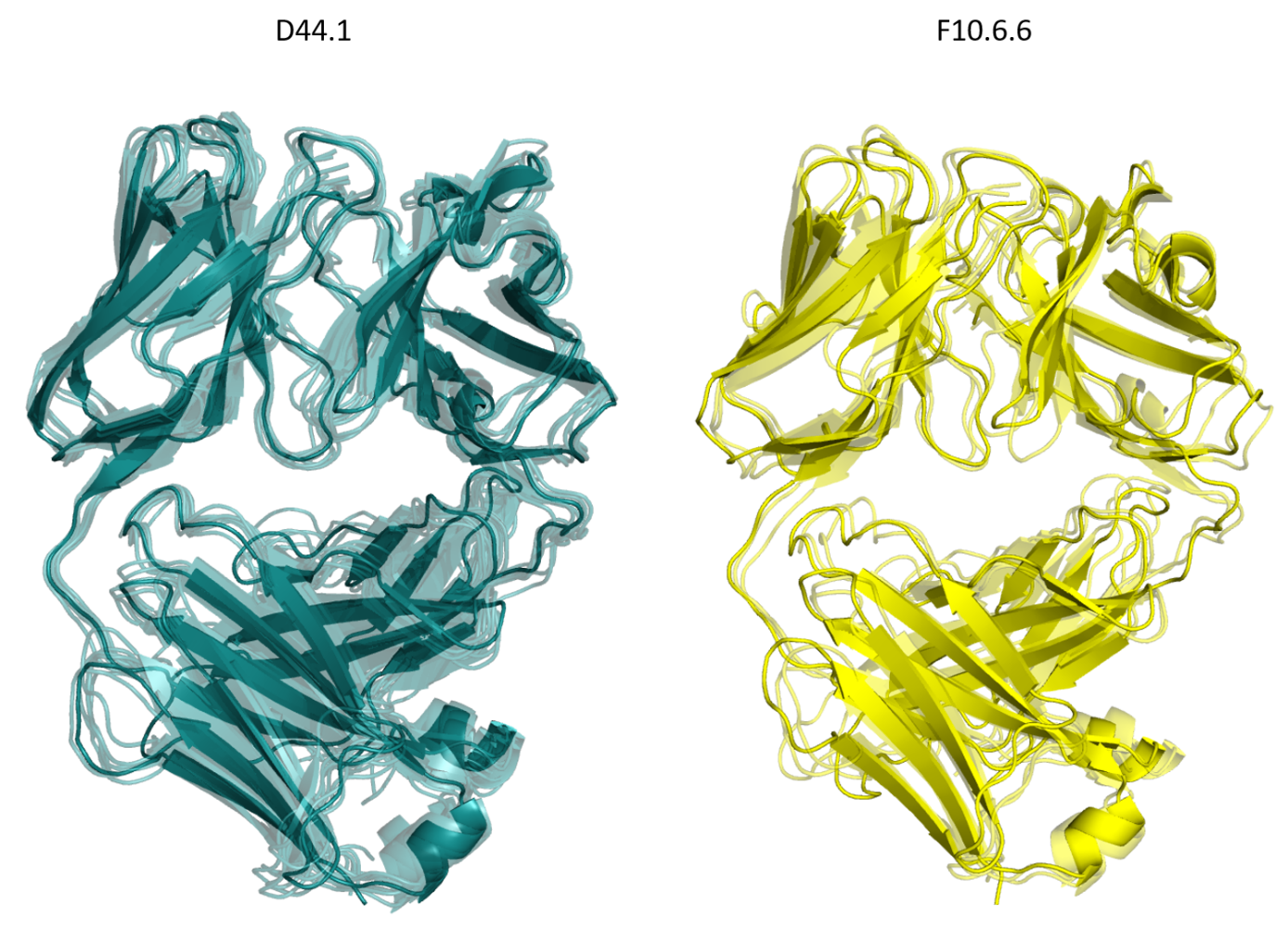


SI Figure S2: Conformational ensemble of the naive D44.1 antibody compared to the matured F10.6.6 antibody. The results clearly show that the overall conformational diversity substantially decreases as a consequence of affinity maturation.


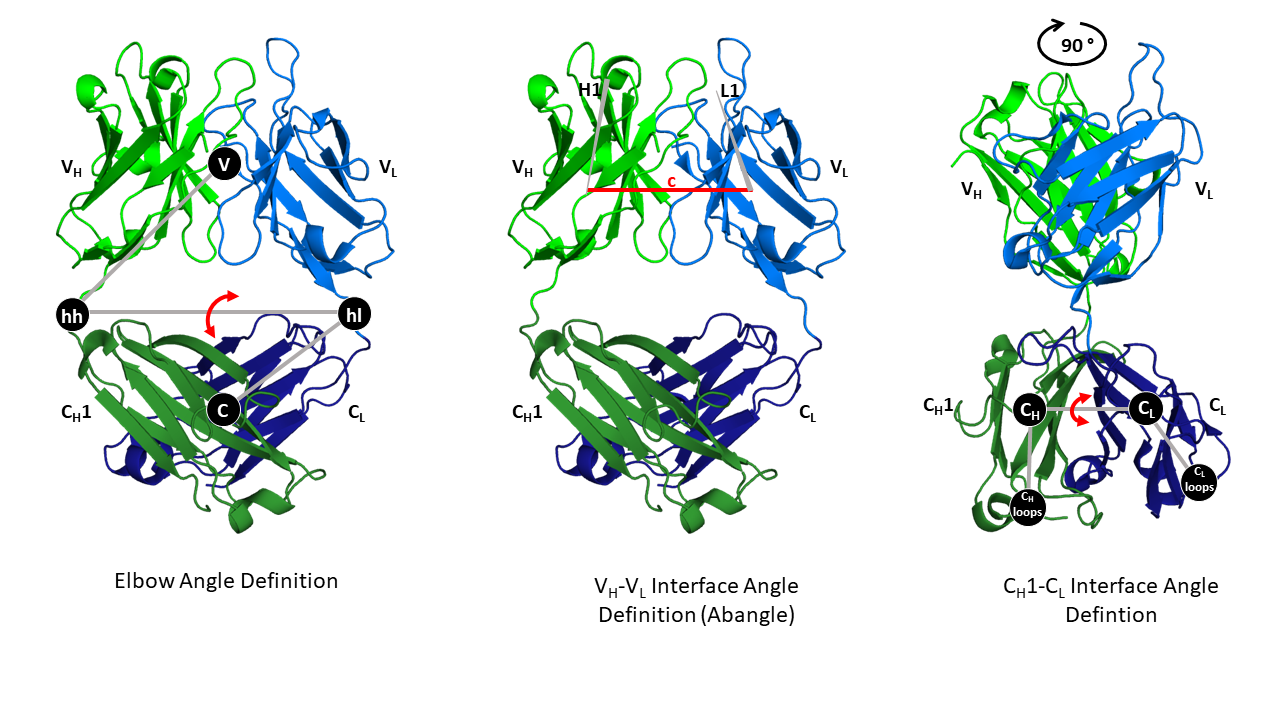


SI Figure S3: Elbow angle, ABangle (HL angle) and C_H_1-C_L_ interface angle definitions projected onto a representative Fab X-ray structure. The constant domains are illustrated in dark green (C_H_1) and dark blue (C_L_), while the variable domains are shown in light green (V_H_) and light blue (V_L_). To calculate the C_H_1-C_L_ interface angle we defined a torsion angle between the center of mass of the C-terminal loops of the C_H_1 domain, the center of mass of the C_H_1, the center of mass of the C_L_ domain and the center of mass of the C-terminal loops of the C_L_ domains. The center of masses of the C_H_1 and C_L_ C-terminal loops are depicted in Figure 1, as C_L_ and C_H_1 loops. For the elbow angle definition all used center of masses to define the torsion angle are illustrated. The center of masses of the switch or hinge regions are abbreviated with hh (hinge heavy) and hl (hinge light).


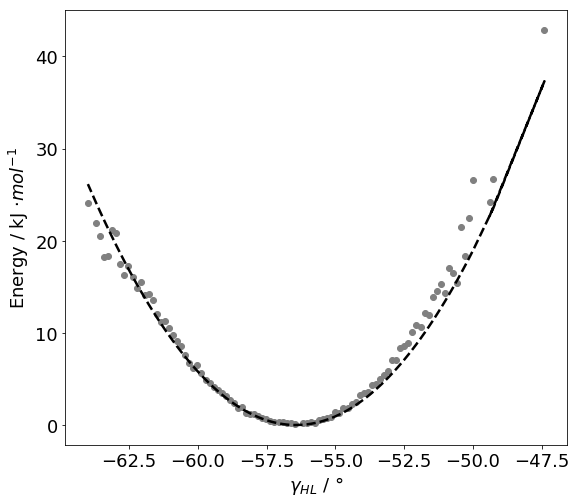


SI Figure S4: An exemplary free energy surface of the 7FAB antibody with the fitted quadratic function is illustrated and shows that these interdomain and elbow angle fluctuations interconvert with each other in the 0.1 to 10 GHz timescale. We estimated the force constants k and included the respective equations used for the frequency f calculations by using the same equations as are shown in Figure 6.
